# Predicting the risk of acute kidney injury after hematopoietic stem cell transplantation: development of a new predictive nomogram

**DOI:** 10.1038/s41598-022-19059-x

**Published:** 2022-09-12

**Authors:** Zhaoping Gan, Liyi Chen, Meiqing Wu, Lianjin Liu, Lingling Shi, Qiaochuan Li, Zhongming Zhang, Yongrong Lai

**Affiliations:** 1 Department of Hematology, Guangxi Medical University First Affiliated Hospital, Nanning, Guangxi China; 2 Spine and Osteopathy Ward, Guangxi Medical University First Affiliated Hospital, Nanning, Guangxi China

**Keywords:** Stem cells, Haematological diseases

## Abstract

The purpose was to predict the risk of acute kidney injury (AKI) within 100 days after hematopoietic stem cell transplantation (HSCT) in patients with hematologic disease by using a new predictive nomogram. Collect clinical data of patients with hematologic disease undergoing HSCT in our hospital from August 2012 to March 2018. Parameters with non-zero coefficients were selected by the Least Absolute Selection Operator (LASSO). Then these parameters were selected to build a new predictive nomogram model. Receiver operating characteristic (ROC) curve, calibration curve, C-index, and decision curve analysis (DCA) were used for the validation of the evaluation model. Finally, the nomogram was further evaluated by internal verification. According to 2012 Kidney Disease Improving Global Guidelines (KDIGO) diagnostic criteria, among 144 patients, the occurrence of AKI within 100 days after HSCT The rate was 29.2% (42/144). The C-index of the nomogram was 0.842. The C-value calculated by the internal verification was 0.809. The AUC was 0.842, and The DCA range of the predicted nomogram was from 0.01 to 0.71. This article established a high-precision nomogram for the first time for predicting the risk of AKI within 100 days after HSCT in patients with hematologic diseases. The nomogram had good clinical validity and reliability. For clinicians, it was very important to prevent AKI after HSCT.

## Introduction

HSCT is the treatment of hematological malignant and benign diseases (such as leukemia, severe Mediterranean Anemia, etc.) effective measures^1^. AKI is one of the common complications after HSCT. The incidence of AKI after HSCT is 20% to 73% and the median time is 31 days. About 4.8% of patients require renal replacement therapy (RRT)^2–4^. As the severity of renal failure increases, the mortality rate of AKI patients also increases, accounting for more than 80% of patients requiring RRT^5^. The incidence of non-recurring deaths in AKI patients is high (HR 2.77, 95% CI 1.8–4.27)^6^. The study found that the cumulative mortality rate of the non-malignant disease AKI group within 100 days after HSCT was significantly higher than that of the non-AKI group (44.4% vs. 0%, p = 0.003)^7^. Therefore, it is of great significance to pay attention to the characteristics of patients with HSCT and establish a prediction of the risk of early AKI. The etiology and risk factors of AKI are complex. It is not only affected by the underlying diseases of transplant patients (such as chronic kidney disease, diabetes, hypertension, etc.), but also related to the medications used before and after transplantation, radiation, and transplant-related complications (such as conditioning toxicity, acute graft versus host disease (aGVHD), infection, hemorrhagic cystitis (HC), conditioning toxicity, etc.^8^.

This study aims to construct a nomogram model to predict the occurrence of AKI within 100 days after HSCT in patients with hematologic diseases. At present, the nomogram model is used to predict the survival period of moderate-risk acute myeloid leukemia, help identify patients with poor prognosis, and guide clinical treatment^9^, predict the risk of severe bleeding in patients with HLA-DQB1 mismatched HSCT^10^. And to predict the prognosis of multiple myeloma patients with pleural effusion^11^. This article reports for the first time a new predictive nomogram to predict the risk of AKI within 100 days after HSCT, which is of great significance for optimizing the management of patients after HSCT and preventing AKI.

## Methods

### Patients

This study was approved by the Ethics Committee of the First Affiliated Hospital of Guangxi Medical University. Collect clinical data of hematologic diseases patients receiving HSCT (August 2012 to March 2018) in our hospital. The criteria for the inclusion of patient data were as follows: (1) The diagnosis was clear and the data were complete; (2) Patients without renal dysfunction before HSCT (defined as glomerular filtration rate (eGFR) ≥ 60 ml/min/1.73 m^2^); (3) The patient received HSCT treatment for the first time. Exclusion criteria: (1) patients with unclear diagnosis and incomplete data; (2) patients with renal impairment before HSCT; (3) The patient received HSCT multiple times.

### Data collection

This study collected clinical data of hematologic diseases patients who underwent HSCT, including gender, age, weight, basic creatinine level, stem cell source, donor source, human leukocyte antigen (HLA) and blood type matching, total body irradiation (TBI), cyclosporin A(CSA), Tacrolimus (FK506), vancomycin, amphotericin B, triazole antifungals, ganciclovir, aGVHD, HC, engraftment syndrome, secondary hypertension, secondary diabetes, infection. The patients were divided into two groups, the AKI group, and the non-AKI group. The criteria for infection were that the patient must had an axillary temperature above 37.7 °C for 1 h or an axillary temperature above 38.0 °C as previously reported^12^. In addition, we also referred to previous literature reports for the standard of aGVHD grade (Table 1)^13,14^. In this study, no patients were diagnosed with TMA. We used peripheral or bone marrow hematopoietic stem cells for patient transplantation.

**Table 1:** Modified aGVHD Glucksberg grading scale.

Grade	Cumulative organ		
	Skin	Liver–bilirubin μmol/L (mg/dl)	Gastrointestinal—amount of diarrhea
1	Rash area < 25%	34–50 (2–2.9)	500–1000 ml/d or pathologically confirmed upper gastrointestinal GVHD
2	Rash area 25–50%	51–102 (3–6)	1000–1500 ml/d
3	Rash area > 50%, body erythema	103–255 (6.1–15)	1500–2000 ml/d
4	Generalized erythema with blister formation or exfoliation	> 255 (> 15)	> 2000 ml/d or severe abdominal pain with + intestinal obstruction
**Degree**			
0 (no)	0	0	0
I (light)	1–2	0	0
II (medium)	1–3	or 1	or 1
III (serious)	2–3	2–3	or 2–4
IV (fatal)	4	or 4	–

R software (Vienna, Austria, https://www.R-project.org) was used for data analysis. The LASSO method was used for all clinical data analysis. Parameters with non-zero coefficients were selected to establish a predictive model. The nomogram was based on a score for each parameter, and the scores for each parameter were added together to obtain a total score. Each total score corresponded to the probability of an outcome event occurring. The discriminative ability and prediction accuracy of the nomogram were evaluated by C-index. The range of the C-index was usually < 0.5, 0.5–0.7, 0.7–0.9, and > 0.9, which represent low accuracy, medium accuracy, high accuracy, and extreme accuracy, respectively^15^. The calibration curve was used to evaluate the actual risk and predicted risk of the AKI nomogram. The predictive ability of the nomogram was evaluated by the AUC curve. The clinical net benefit was evaluated the DCA curve. Finally, we chose an internal verification method to verify the nomogram. The C-index was calculated through the bootstrapping verification of the AKI nomogram (1000 bootstraps resamples).

### Ethics approval and consent to participate

All procedures were performed following relevant guidelines. This paper has been approved by the ethics committee of The First Affiliated Hospital of Guangxi Medical University. Written informed consent of patients has been obtained for this study.

## Results

### Patients’ characteristics

A total of 144 patients with hematological diseases who underwent HSCT were collected from August 2012 to March 2018 in our hospital, including 66 males and 78 females. Among all patients, 42 cases developed AKI and 102 cases without AKI. The data of the two groups were shown in Table 2, including the general data of the patients, transplant characteristics, drugs used, postoperative complications, and other information. In this study, a total of 42 patients with AKI were included for analysis, including 11 with stage I, 16 with stage II, and 15 with stage III renal impairment.

**Table 2:** Comparison of clinical data between AKI group and non-AKI group.

	Non-AKI group (N = 102)	AKI group (N = 42)	P-value
**Gender**			
Female	52 (51%)	17 (40%)	0.335
Male	50 (49%)	25 (60%)	
**Age**			
< 20y	14 (14%)	7 (17%)	0.747
20–50y	77 (75%)	32 (76%)	
> 50y	11 (11%)	3 (7%)	
**Weight**			
≤ 50 kg	69 (68%)	29 (69%)	1
> 50 kg	33 (32%)	13 (31%)	
**FK506**			
No	85 (83%)	23 (55%)	< 0.001**
Yes	17 (17%)	19 (45%)	
**Donor source**			
Auto	21 (21%)	0 (0%)	0.003*
Allo	81 (79%)	42 (100%)	
**Stem cell source**			
PB	26 (25%)	13 (31%)	0.643
PB + BM	76 (75%)	29 (69%)	
**HLA**			
Mismatch	9 (9%)	14 (33%)	< 0.001**
Match	93 (91%)	28 (67%)	
**Blood type matching**			
No	28 (27%)	25 (60%)	< 0.001**
Yes	74 (73%)	17 (40%)	
**aGVHD**			
No	83 (81%)	21 (50%)	< 0.001**
Yes	19 (19%)	21 (50%)	
**HC**			
No	90 (88%)	35 (83%)	0.604
Yes	12 (12%)	7 (17%)	
**Vancomycin**			
No	46 (45%)	17 (40%)	0.746
Yes	56 (55%)	25 (60%)	
**Sepsis**			
No	94 (92%)	37 (88%)	0.65
Yes	8 (8%)	5 (12%)	
**Infection**			
No	33 (32%)	2 (5%)	< 0.001**
Yes	69 (68%)	40 (95%)	
**Ganciclovir**			
No	29 (28%)	9 (21%)	0.51
Yes	73 (72%)	33 (79%)	
**TBI**			
No	77 (75%)	28 (67%)	0.381
Yes	25 (25%)	14 (33%)	
**CSA**			
No	44 (43%)	21 (50%)	0.57
Yes	58 (57%)	21 (50%)	
**Triazole antifungals**			
No	24 (24%)	5 (12%)	0.176
Yes	78 (76%)	37 (88%)	
**Amphotericin B**			
No	100 (98%)	40 (95%)	0.71
Yes	2 (2%)	2 (5%)	
**Implantation syndrome**			
No	99 (97%)	41 (98%)	1
Yes	3 (3%)	1 (2%)	
**Secondary diabetes**			
No	100 (98%)	39 (93%)	0.297
Yes	2 (2%)	3 (7%)	
**Secondary hypertension**			
No	89 (87%)	38 (90%)	0.795
Yes	13 (13%)	4 (10%)	
**Cytomegaloviremia**			
No	78 (76%)	24 (57%)	0.034*
Yes	24 (24%)	18 (43%)	
**Basal creatinine**			
44–106 μmol/L	87 (85%)	33 (79%)	0.461
< 44 μmol/L	15 (15%)	9 (21%)	

Tacrolimus (FK506), acute graft versus host disease (aGVHD), hemorrhagic cystitis (HC), total body irradiation (TBI), cyclosporin A(CSA).

*P < 0.05, **P < 0.01.

**Figure 1:**
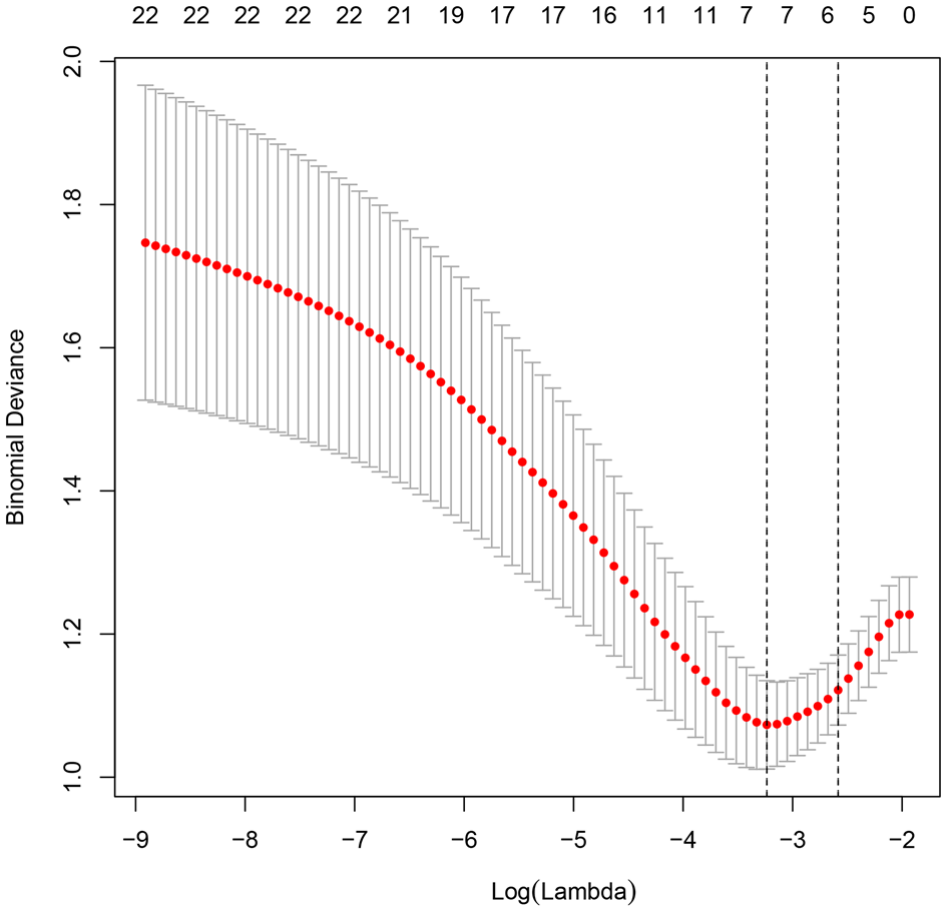
The LASSO model was constructed to select the optimal parameters (lambda) and the relationship graph between binomial deviance and log (lambda) was drawn.

**Figure 2:**
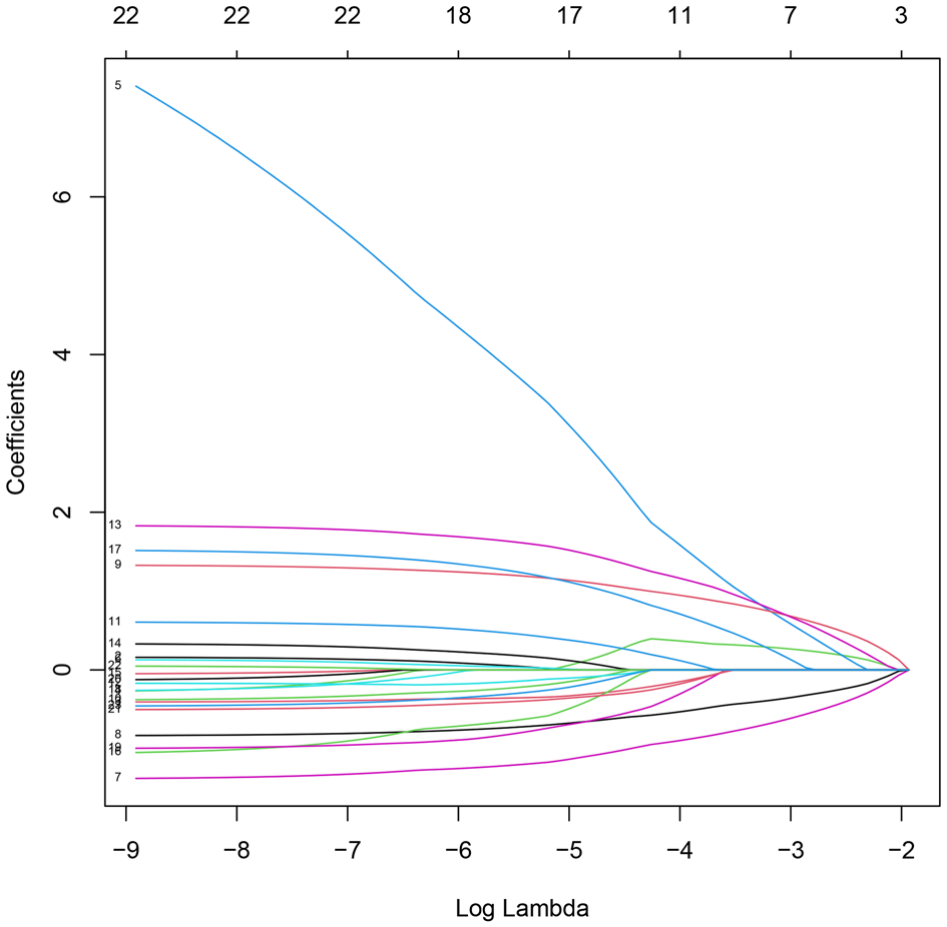
The features with nonzero coefficients were selected by optimal lambda.

**Figure 3:**
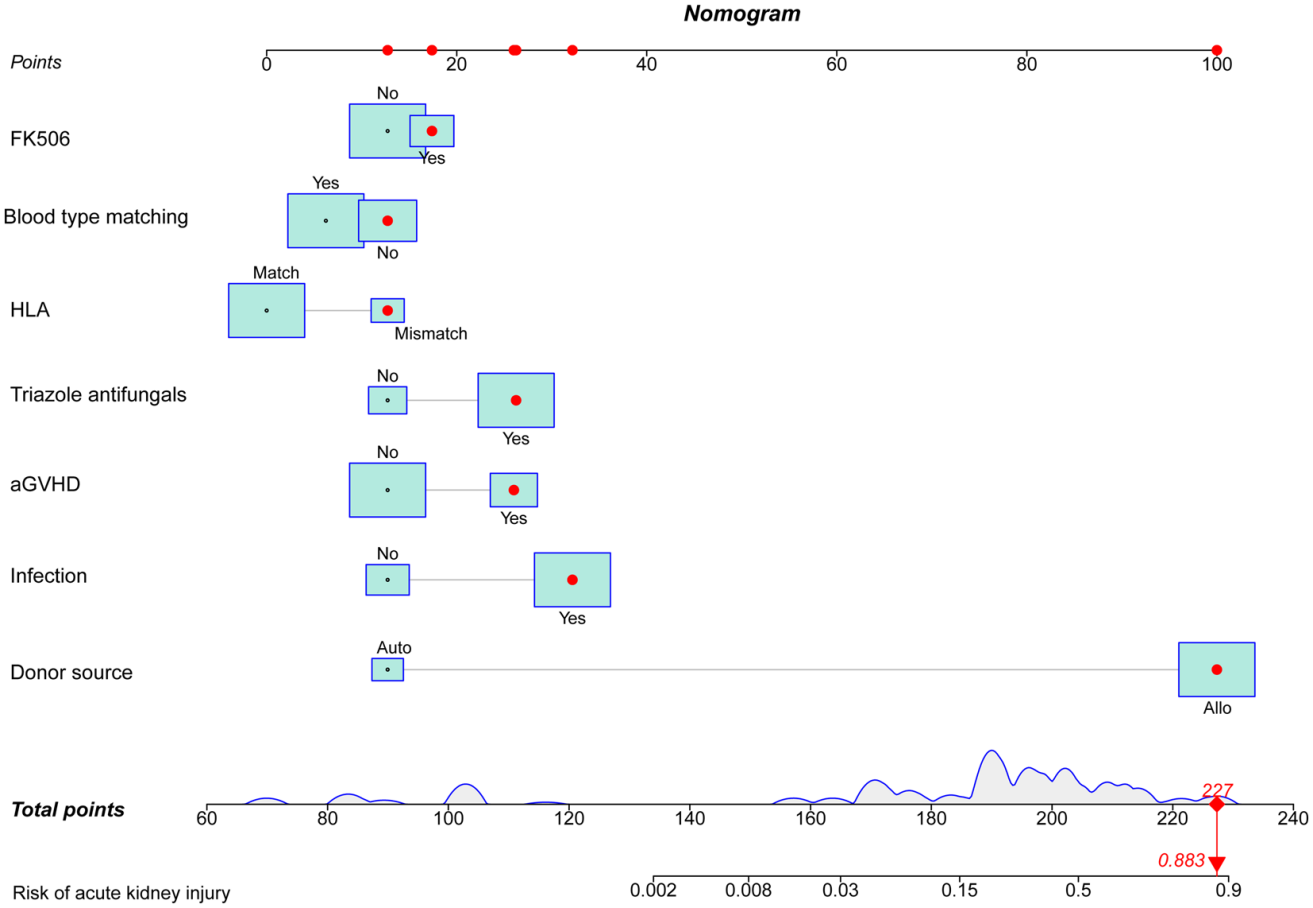
The nomogram was constructed to predict the risk of AKI.

All data were analyzed by the LASSO regression analysis. A total of 7 parameters with non-zero coefficients were obtained. The binomial deviation (Fig. 1) and coefficient (Fig. 2) were obtained under the optimal lambda by LASSO analysis. A new nomogram was constructed to predict the risk of AKI within 100 days after HSCT (Fig. 3). The red dot for each parameter was specific information for that patient. The total score obtained for this patient was 227 points, and the predicted probability of AKI was 88.3% (Fig. 3).

**Figure 4:**
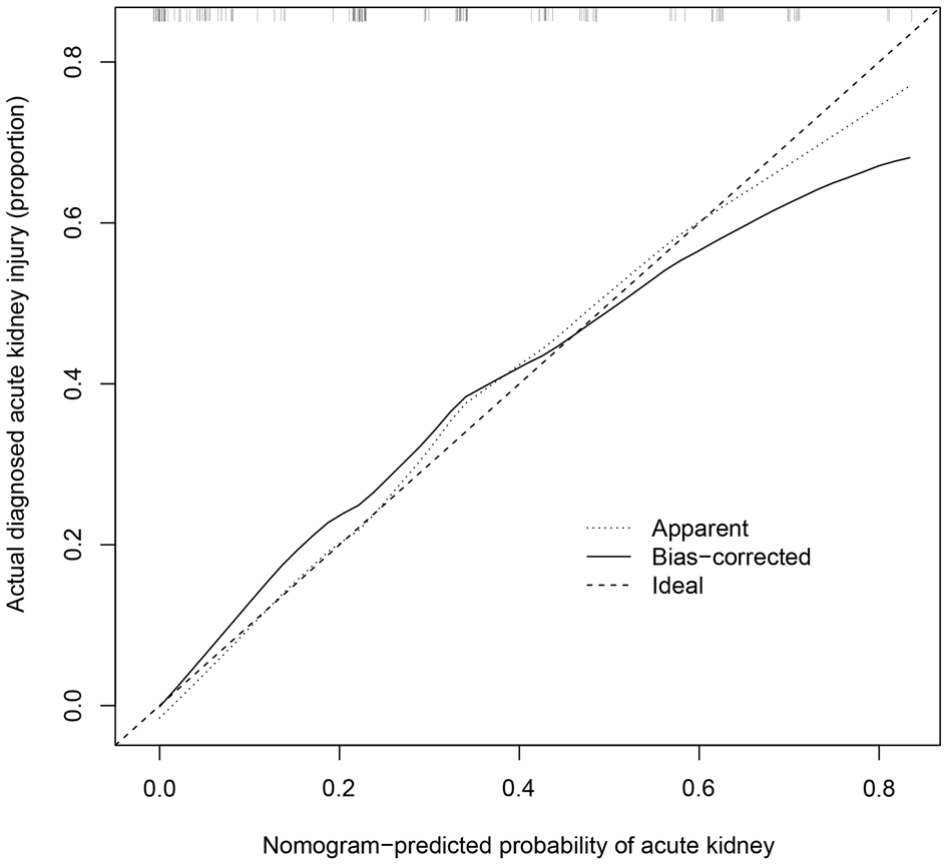
Calibration curves for the nomogram of AKI.

**Figure 5:**
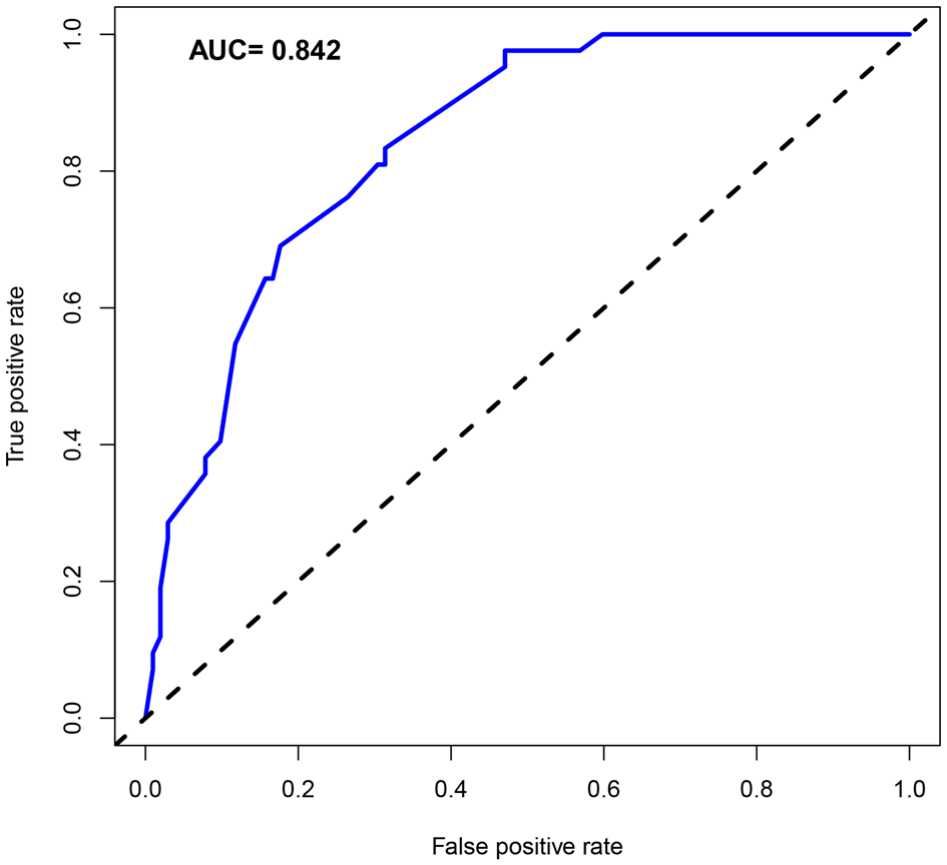
Receiver operating characteristic curve of the prediction nomogram. The AUC of the nomogram scoring system for predicting AKI was 0.842.

**Figure 6:**
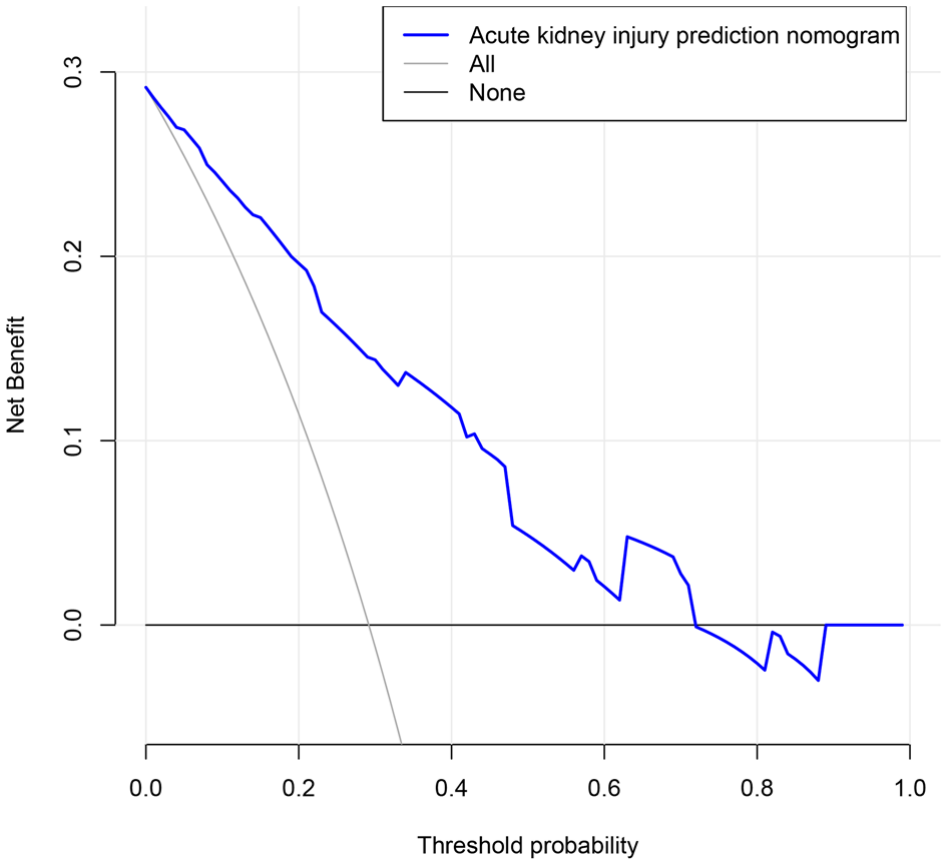
The nomogram of the decision curve analysis and the net benefit of the model ranged from 0.01 to 0.71.

The C-index was measured to evaluate the predictive ability of the new nomogram, and the C-index was 0.842. The calibration curve was close to the ideal curve, indicating that the model had a good predictive ability (Fig. 4). The ROC curve was further constructed, and the AUC was calculated to be 0.842 (Fig. 5). The net benefit of the predictive nomogram ranged from 0.01 to 0.71, which was determined by DCA curve (Fig. 6).

To further verify the actual predictive ability and stability of the nomogram. we chose an internal verification method. The C-index was analyzed by the bootstrap verification of the nomogram (1,000 bootstrap resampling). The C-index was 0.809, which was a very close C-index of 0.842 for the training set.

## Discussion

AKI is a common complication after HSCT. The increased severity of AKI was associated with an increased risk of death^7^. The pathogenesis of AKI after HSCT was complicated and affected by many factors. Prerenal azotemia was a common cause of AKI in HSCT patients. Common adverse reactions to chemotherapy include nausea, vomiting, diarrhea, and mucositis, which often caused excessive fluid loss through the gastrointestinal tract or insufficient oral intake to cause circulation blood volume to be reduced, which eventually resulted in prerenal kidney injury^16,17^. In addition, acute tubular necrosis was also a common cause of AKI after HSCT. Hypovolemic shock, septic shock, or nephrotoxic drugs, such as amphotericin B, vancomycin and CSA could cause acute renal tubular necrosis and cause renal AKI, which could cause AKI alone or in synergy with prerenal etiology^16–18^. Urinary obstruction might be the cause of AKI in patients with HSCT. Intravenous infusion of ganciclovir and other antiviral drugs could precipitate in the urine and form crystals in the renal tubules, causing obstruction, and blood clots formed by HC could lead to urinary tract obstruction, resulting in obstructive postrenal AKI^19^. Although these complications might not be independent risk factors for AKI, their combination might lead to the occurrence of AKI. In addition, nephrotoxic drugs used to treat these complications could also cause AKI^20^.In addition, hypertension and diabetes also lead to the occurrence of AKI after HSCT^3,18^. Several risk factors for AKI in patients undergoing HSCT had been reported. The descriptions of transplant characteristics, such as donor, race, TBI, nephrotoxic agents, and post-transplant adverse events, such as aGVHD and infection, were inconsistently described as risk factors for the development of AKI^21–23^. Studies had shown that unrelated donors were closely associated with AKI (HR, 6.26; P = 0.042)^20^.It had been reported that transplantation of hematopoietic stem cells from unrelated donors was associated with a significant increase in the risk of infection, severe aGVHD, and organ toxicity^24–29^. Calcineurin inhibitor (CNIs) caused AKI by arteriolar vasoconstriction, reducing kidney perfusion, tubular toxicity, and endothelial injury^30,31^. CNIs nephrotoxicity after hematopoietic transplantation was reported in up to 31% of patients^32^. The infection could lead to hemodynamic changes and inflammatory damage, leading to AKI. The infection resulted in systemic arteriole constriction and endothelial damage, causing capillary leakage and renal insufficiency^33^. Damage to the tubules themselves led to the release of local cytokines and chemokines, resulting in local inflammation and further intrarerenal damage^34^. In addition, antimicrobials commonly used to treat infection were often nephrotoxic. However, the mechanism of AKI induced by triazole antifungals remained unclear^35^. Liu et al. found that HLA mismatched was closely related to AKI after HSCT (OR = 3.6; 95%CI = 1.1–13.0)^36^. And ABO mismatched was found to be associated with a significantly increased risk of grade II-IV aGVHD^37^. This might be the reason why ABO mismatched were associated with AKI.

AKI after HSCT could seriously affect the survival and prognosis of patients, so it was important to identify the risk of AKI in advance. At present, studies were using the hematopoietic cell transplantation-specific comorbidity index (HCT-CI) to study the incidence of AKI after allogeneic HSCT. HCT-CI factors included high disease risk, related donor, myeloablative conditioning regimen, stem cell source, prior stem cell transplant^38^. At present, this study was the first time to use the nomogram model to predict the risk of AKI within 100 days after HSCT.

In this paper, we introduced perioperative parameters to develop the nomogram of predicting AKI risk. The C-index was 0.842 and 0.809 in the training and validation set respectively, which revealed that the prediction ability of the nomogram was characterized by high accuracy^15^. The study showed that the higher the C-index of internal validation, the better the efficiency of identification and comparison^15^. In addition, we performed the decision curve analysis to estimate the actual clinical benefit of the nomogram. The results of DCA also showed high validity and predictive effect. The risk of AKI after HSCT could be predicted in advance by using the nomograph model, which could guide the management of HSCT patients, improve the survival of patients and improve the quality of life of patients. The results of this study showed that the factors included in predicting the risk of AKI after HSCT were donor, HLA, blood type matching, FK506, aGVHD, infection and triazole antifungal drugs.

However, this paper had some limitations. (1) This study was a retrospective analysis without a prospective study. (2) More cases need to be added to verify the nomogram.

## Conclusion

In the article, the nomogram had good clinical validity and reliability. We found some factors that can be used to predict the risk of AKI within 100 days after HSCT, including donor source, HLA, blood type matching, FK506, aGVHD, infection, and triazole antifungal drugs. The nomogram can be used to predict the risk of AKI to optimize patient management during the diagnosis and treatment of HSCT.

## Data Availability

The datasets used and/or analysed during the current study available from the corresponding author on reasonable request.
